# HIV Modulates Osteoblast Differentiation via Upregulation of RANKL and Vitronectin

**DOI:** 10.3390/pathogens13090800

**Published:** 2024-09-15

**Authors:** Rosa Nicole Freiberger, Cynthia Alicia Marcela López, María Belén Palma, Cintia Cevallos, Franco Agustin Sviercz, Patricio Jarmoluk, Marcela Nilda García, Jorge Quarleri, M. Victoria Delpino

**Affiliations:** 1 Laboratorio de Inmunopatología Viral, Instituto de Investigaciones Biomédicas en Retrovirus y Sida (INBIRS), Universidad de Buenos Aires, Consejo de Investigaciones Científicas y Técnicas (CONICET), Buenos Aires 1121, Argentina; freibergernicole@gmail.com (R.N.F.); alilopez1996@gmail.com (C.A.M.L.); cevalloscintia@gmail.com (C.C.); patriciojarmoluk@gmail.com (P.J.); quarleri@fmed.uba.ar (J.Q.); 2 Cátedra de Citología, Histología y Embriología, Facultad de Ciencias Médicas, Universidad Nacional de La Plata, La Plata 1900, Argentina; 3 Laboratorio de Investigación Aplicada a Neurociencias (LIAN), Fleni, Consejo de Investigaciones Científicas y Técnicas (CONICET), Escobar 1625, Argentina

**Keywords:** osteoblasts, HIV, bone, vitronectin

## Abstract

Bone loss is a prevalent characteristic among people with HIV (PWH). We focused on mesenchymal stem cells (MSCs) and osteoblasts, examining their susceptibility to different HIV strains (R5- and X4-tropic) and the subsequent effects on bone tissue homeostasis. Our findings suggest that MSCs and osteoblasts are susceptible to R5- and X4-tropic HIV but do not support productive HIV replication. HIV exposure during the osteoblast differentiation process revealed that the virus could not alter mineral and organic matrix deposition. However, the reduction in runt-related transcription factor 2 (RUNX2) transcription, the increase in the transcription of nuclear receptor activator ligand kappa B (RANKL), and the augmentation of vitronectin deposition strongly suggested that X4- and R5-HIV could affect bone homeostasis. This study highlights the HIV ability to alter MSCs’ differentiation into osteoblasts, critical for maintaining bone and adipose tissue homeostasis and function.

## 1. Introduction

Individuals that have acquired human immunodeficiency virus (HIV) infection often experience a significant decrease in bone density, rendering them more susceptible to fractures [1]. Although antiretroviral medication has significantly extended patient survival, it has also led to the emergence of long-term side effects, including bone abnormalities. The deterioration of bone health in PWH can be attributed to several factors, such as the use of antiretroviral medication, patient lifestyle choices, and overall health factors like age, body mass index, or muscle wasting [2,3,4]. Furthermore, bone deficiencies in untreated individuals strongly suggest that the virus independently plays a role in this process [5,6,7,8]. However, the effect of antiretroviral medication on bone mineral density (BMD) varies with the type of treatment. Low BMD has been associated with nucleoside analog reverse-transcriptase inhibitors (NRTIs) [9,10,11]. Specifically, tenofovir disoproxil fumarate (TDF) has been linked to greater BMD loss compared to other regimens, such as lamivudine (3TC) or emtricitabine (FTC) or two-drug combinations [4,9,10,11,12,13,14,15]. However, some studies report conflicting findings on TDF’s impact on BMD over time [16].

The skeleton is a dynamic system continually undergoing remodeling, driven by the activity of bone-resorbing osteoclasts and bone-forming osteoblasts. Imbalances between these osteoclasts and osteoblasts can result in bone abnormalities [17,18,19]. Osteoclasts primarily originate from the fusion of monocyte/macrophage precursors derived from hematopoietic stem cells, while osteoblasts arise from cells of mesenchymal origin. This distinction in their origin plays a crucial role in bone remodeling, maintaining the balance between bone formation and resorption in the skeletal system [20,21]. Osteoblasts play a crucial role in depositing the bone matrix and are essential for bone calcification and mineralization [6]. In contrast, osteoclasts are responsible for bone resorption through acidification and the release of lysosomal enzymes [7].

Multipotent mesenchymal stem cells (MSCs) found in the bone marrow originate osteoblasts. In addition, these MSCs can differentiate into other various cell types, including fibroblasts, myocytes, chondrocytes, and adipocytes. The differentiation process depends on a variety of external cues that contribute to the delicate balance of determining cell fate [22].

The formation of bone tissue and its homeostatic maintenance is largely attributed to the activity of MSCs residing in the bone marrow. The microenvironment within the bone marrow plays a critical role in maintaining MSCs and regulating the osteogenic process, influenced by signals from systemic factors and the extracellular matrix. In this context, the differentiation of MSCs into adipocytes in healthy bone tissue is tightly regulated [23].

Due to the expression of CD4 receptors and CCR5 and CXCR4 coreceptors, MSCs are believed to be susceptible to HIV infection [24], although productive infection has not been observed until now [24]. 

In vitro studies revealed that Tat and Nef proteins cumulatively reduce the number of bone marrow MSCs available for osteoblast differentiation by inducing early senescence, oxidative stress, and mitochondrial dysfunction. Additionally, Tat increases NF-κB activity and cytokine secretion, while Nef inhibits early autophagy, with no effect from Tat on autophagy [17]. On the other hand, p55-Gag reduces osteogenesis by impacting MSC differentiation into osteoblasts, while Rev induces an increase in osteogenesis [25].

In the present study, we investigate whether HIV could infect osteoblasts and their precursors (MSCs), as well as the impact of HIV on osteoblastogenesis.

## 2. Materials and Methods

### 2.1. Isolation and Expansion MSCs

Umbilical cords were preserved in α-Minimal Essential Medium (α-MEM, Gibco, Life Technologies, Grand Island, NY, USA) and processed as described previously [26]. In summary, each cord was cut into 5 mm fragments and a sagittal incision exposed Wharton’s jelly. The careful removal of umbilical blood vessels was performed using clamps. Fragments were then washed 2 or 3 times with Dulbecco’s phosphate-buffered saline from Sigma-Aldrich to eliminate residual blood. The section with the exposed jelly was placed face down at the bottom of a culture plate and α-MEM with 10% platelet lysate was added. Plates were incubated at 37 °C in a humid atmosphere containing 5% CO_2_, with the cell culture medium replenished every 2 to 3 days. The expansion of umbilical-cord-derived MSCs was typically observed between 10 and 14 days after explantation, and cells were further amplified until reaching passage 2/3. MSCs were characterized by the expression of CD105, CD73, and CD90 and the absence of CD45, CD34, CD14, CD19, and HLA-DR molecules [27]. For experiments, MSCs were grown in α-MEM supplemented with 10% heat-inactivated fetal bovine serum (Gibco), 100 U/mL of penicillin, and 100 mg/mL of streptomycin (complete medium) and were used until passage 5. This study received approval from the Comité de Bioética y Ética de la Facultad de Ciencias Médicas de la Universidad de Buenos Aires, Argentina (RESCD-2023-1291). Written informed consent was obtained from each mother before normal cesarean birth, and human umbilical cords were obtained from discarded placentas.

### 2.2. Infection of Mesenchymal Stem Cells and Osteoblasts by Cell-Free Wild-Type HIV-X4 or R5-Tropic

The full-length infectious clones of wild-type (WT)-HIV AD8 (R5-tropic) and NL43 (X4-tropic) strains [28,29] were obtained from the NIH AIDS Reagent Program (Division of AIDS, NIAID, NIH, Bethesda, Maryland, USA). The quantification of HIV capsid (p24 antigen) in viral stocks was conducted using a commercial ELISA assay (INNOTEST^®^ HIV Antigen mAb, Fujirebio, Tokio, Japan).

### 2.3. Osteoblast Differentiation

MSCs were initially seeded at a density of 5 × 10^4^ cells per well in 24-well plates and cultured until reaching confluence using a complete medium. Following this, the medium was substituted with osteoblast differentiation medium, consisting of complete media enriched with 10 mM β-glycerophosphate, 0.1 µM dexamethasone, and 50 µM ascorbic acid, all sourced from Sigma-Aldrich (Burlington, MA, USA). Complete differentiation was achieved by day 14–21.

### 2.4. CD4, CXCR4, and CCR5 Surface Expression

At 0 (MSCs), 7, and 14 days of the osteoblast differentiation process, the cells were washed and subjected to staining for surface antigens. This staining procedure involved incubation with the following antibodies for 30 min at 4 °C: FITC Mouse anti-human CD195 (BD PharmingenTM, San Diego, CA, USA), PE Mouse Anti-Human CD184 (BD PharmingenTM, USA), and PerCP anti-human CD4 antibody (Biolegend, San Diego, CA, USA). Data were acquired using a Full-Spectrum Flow Cytometry Cytek^®^ Northern Lights 3000™ (Cytek Biosciences Inc., Fremont, CA, USA) and analyzed with FlowJo.v10.6.2 (Ashland, OH, USA).

### 2.5. HIV Infections Using X4 and R5-Tropic Strains

MSCs and differentiated osteoblasts (14 days) were infected with an inoculum of 1 pg of p24/cell with the CCR5 tropic HIV strain AD8 and, separately, with the CXCR4 tropic HIV strain NL43, following the procedure described previously [30]. Infection efficiency was assessed by measuring intracellular p24 expression using the KC57 monoclonal antibody labeled with phycoerythrin against p24 (PE-KC57 (FH190-1-1)) protein (Beckman Coulter, Brea, CA, USA) by confocal microscopy using a Zeiss LSM 800 confocal microscope (Zeiss, Oberkochen, Germany).

The detection of HIV proviral DNA was conducted using the Alu-PCR technique, as described by Kumar et al. [31]. MSCs and osteoblast cells exposed to HIV, along with ACH-2 cells (positive control), were centrifuged in 1.5 mL microtubes at 16,000× *g* for 5 min. After carefully removing and discarding the supernatants, cell pellets were resuspended in lysis buffer (10 mM Tris-HCl, pH 8.0, 50 nM KCl, 400 µg/mL proteinase K; Invitrogen, Waltham, MA, USA) at appropriate concentrations (20 × 10^6^ cells/mL for ACH-2 cells; 5 × 10^6^ to 10 × 10^6^ cells/mL for LX-2) and digested for 12 to 16 h at 55 °C in a heating shaker. Proteinase K was then inactivated by heating at 95 °C for 5 min. The cell lysates were either immediately used for HIV DNA quantification or stored at −70 °C until use.

Integrated HIV DNA determination was performed in a 50 µL reaction mixture containing Taq polymerase buffer (Invitrogen), 3 mM MgCl_2_, 300 mM deoxynucleoside triphosphates (Invitrogen), 300 nM of each of the 4 primers, and 2.5 U Taq polymerase (Invitrogen).

The primers used for preamplification were ULF1-5′-ATG CCA CGT AAG CGA AAC TCT GGG TCT CTC TDG TTA GAC-3′; Alu1-5′-TCC CAG CTA CTG GGG AGG CTG AGG-3′; and Alu2-5′-GCC TCC CAA AGT GCT GGG ATT ACA G-3′. For real-time PCR, the following primers were used: Lambda T-5′-ATG CCA CGT AAG CGA AAC T-3′; UR2-5′-CTG AGG GAT CTC TAG TTA CC-3′; and UHIV TaqMan LC640-5′-CAC TCA AGG CAA GCT TTA TTG AGG C-3′.

### 2.6. Cellular mRNA Preparation and RT-qPCR

Total cellular mRNA was extracted using Quick-RNA MiniPrep Kit (Zymo Research, Irvine, CA, USA) and 1 µg of RNA was employed to perform the reverse transcription using Improm-II Reverse Transcriptase (Promega, Madison, WI, USA). Real-time PCR was conducted with SYBR green as a DNA-binding fluorescent dye using a StepOne Real-Time PCR System (Applied Biosystems, Foster City, CA, USA). The following primer pairs were used: β-actin sense 5-CCTGGCACCCAGCACAAT-3, antisense 5-CGGGATCCACACGGAGTACT-3; RUNX2 sense 5-GGAGTGGACGAGGCAAGAGTTT-3, antisense 5-AGCTTCTGTCTGTGCCTTCTGG-3; and RANKL sense 5-GCCAGTGGGAGATGTTAG-3, antisense 5-TTAGCTGCAAGTTTTCCC-3. All primer sets yielded a single product of the correct size. The amplification cycle for β-actin and RUNX2 was 95 °C for 15 s, 58 °C for 30 s, and 72 °C for 60 s, while, for RANKL, it was 95 °C for 15 s, 54 °C for 30 s, and 72 °C for 60 s. Relative transcript levels were calculated using the 2^−ΔΔCt^ method, normalized by gene β-actin.

### 2.7. RANKL Protein Expression

RANKL was determined in culture supernatants using an ELISA kit (R&D Systems, Minneapolis, MN, USA) according to the manufacturer’s instructions.

### 2.8. Alizarin Red S Staining

To assess calcium deposition in osteoblasts, alizarin red staining was employed. Osteoblast cells were seeded in 24-well plates. After 7, 14, and 21 days of culture differentiation, osteoblasts were fixed in 4% paraformaldehyde (PFA) for 10 min at room temperature. Following fixation, cells were washed with deionized water and stained with 2% (*w*/*v*) alizarin red S. Subsequently, the stained cells were visualized using light microscopy or processed for quantitative analysis.

For quantitative analysis, monolayers were treated with 10% (*v*/*v*) acetic acid added to each well and incubated at room temperature for 30 min with shaking. Following this, cells were detached, heated at 85 °C for 10 min, and centrifuged at 20,000× *g* for 15 min. Supernatants were neutralized by the addition of 10% (*v*/*v*) ammonium hydroxide. Absorbance at 405 nm was measured on a microplate reader (Metertech, Inc., Taipei, Taiwan) against 0.1 N sodium hydroxide as a blank.

### 2.9. Alkaline Phosphatase (ALP) Staining

ALP activity in osteoblasts was assessed after 7, 14, and 21 days of culture differentiation using a solution containing 5-Bromo-4-chloro-3-indolylphosphate (BCIP) and nitroblue tetrazolium (NBT) obtained from Sigma, following the manufacturer’s instructions. Briefly, cells were exposed to the BCIP–NBT substrate in a darkened environment at room temperature for 10 min. The resulting colorimetric reaction was stopped by gently rinsing the cells with distilled water.

For the quantification of ALP activity, cell lysis was performed using a 0.1 M Tris buffer supplemented with 0.5% Triton X-100. Subsequently, cell lysates containing 2 mg of total protein were incubated with p-nitrophenylphosphate (pNPP) at 37 °C for 10 min. The reaction was terminated by the addition of 0.5 M NaOH, and the resulting absorbance at 420 nm was measured using a microplate reader.

### 2.10. Assessment of Collagen Deposition by Sirius Red Staining

Collagen deposition in osteoblasts was evaluated after 7, 14, and 21 days of culture differentiation using Sirius red (Sigma-Aldrich (Burlington, MA, USA). Before fixing with 1 mL of Bouin’s fluid for 1 h, cell layers were thoroughly washed with PBS. After removing the fixation fluid, the culture plates underwent three washes with deionized water and were allowed to air-dry. Subsequently, 1 mL of Sirius red dye reagent, dissolved in saturated aqueous picric acid at a concentration of 0.1% in Bouin’s fluid, was applied. Cells were gently agitated and stained for 18 h. To remove unbound dye, the stained cell layers were extensively washed with 0.01 N hydrochloric acid. Following rinsing, they were analyzed using light microscopy.

For quantitative assessment, the stained material was dissolved in 0.2 mL of 0.1 N sodium hydroxide, achieved by shaking for 30 min. The resulting dye solution was transferred to microtiter plates, and the optical density (OD) was measured at 550 nm against a blank of 0.1 N sodium hydroxide using a microplate reader (Metertech, Inc., Taipei, Taiwan).

### 2.11. Assessment of Vitronectin by Immunofluorescence 

After 14 and 21 days of osteoblast differentiation, cells were fixed with 4% paraformaldehyde at room temperature for 10 min. Subsequently, the osteoblasts underwent an initial incubation with mouse anti-vitronectin antibodies (Abcam, UK), appropriately diluted in PBS Tween 0.025% and 1% of BSA overnight at 4 °C. This was followed by a second incubation with Alexa Fluor 488 anti-mouse antibodies (Abcam). For nuclear counterstaining, 4,6-diamidino-2-phenylindole (DAPI) (Molecular Probes, Eugene, OR, USA) was used. 

The prepared specimens were analyzed using a Nikon Eclipse TI-S L100 microscope (Nikon, Tokyo, Japan).

### 2.12. U937 Adhesion Assay

U937 cell line (American Type Culture Collection, ATCC, Rockville, MD, USA) was cultured in a 5% CO_2_ atmosphere at 37 in RPMI medium supplemented as a complete medium. After 21 days of differentiation, osteoblasts were incubated with 1 × 10^6^ U937 cells previously labeled with CellTrace yellow (Thermo Fisher Scientific, Waltham, MA, USA). After incubating for 2 h at 37 °C, the nonadherent cells were washed away with sterile phosphate-buffered saline (PBS). The adhered U937 cells were visualized using a Nikon Eclipse TI-S L100 microscope, counted in 10 randomly selected visible fields, and quantified using ImageJ.

### 2.13. Statistical Analysis

Where applicable, statistical analysis was performed. Multiple comparisons between all pairs of groups were made with Tukey’s test, and those against two groups were made with the Mann–Whitney U test. Graphical and statistical analyses were performed with GraphPad Prism 5.0 software (GraphPad oftware, San Diego, CA, USA). Each experiment was performed in triplicate with different culture preparations on five independent occasions. Data were represented as mean ± SD. A *p* < 0.01 is represented as *, *p* < 0.001 as **, and *p* < 0.0001 as ***; *p* < 0.01 was the minimum level regarded as a statistically significant difference between groups.

## 3. Results

### 3.1. Cell Membrane Expression of HIV Receptor and Co-Receptors on MSCs and Osteoblasts 

**Figure 1:**
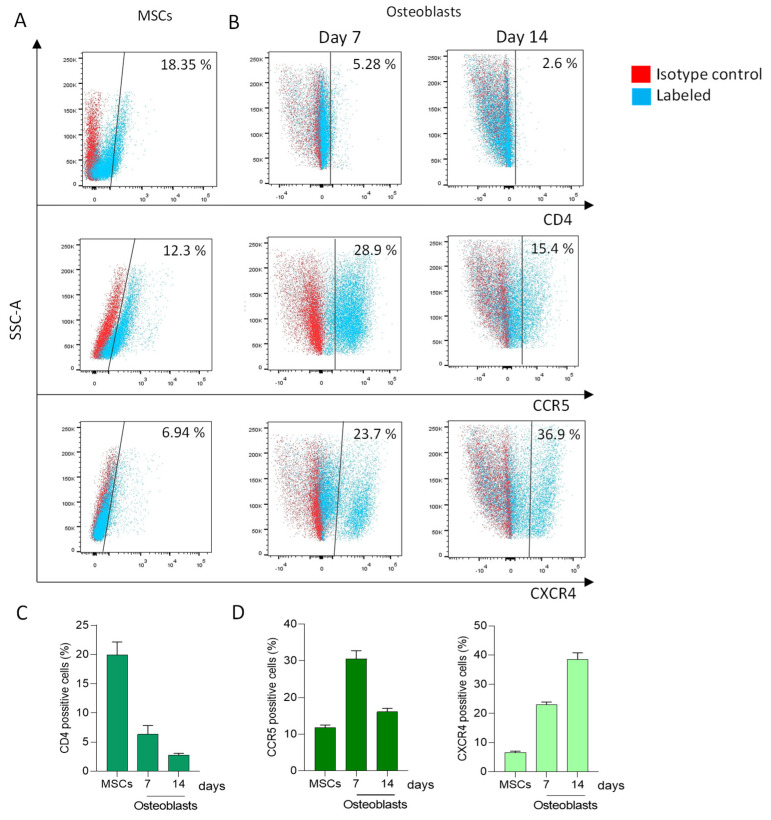
Expression of CD4, CCR5, and CXCR4 on the cell surface. Representative dot plots obtained by flow cytometry indicating the surface marker expression of CD4, CCR5, and CXCR4 in mesenchymal stem cells (MSCs) (**A**), and at 7 and 14 days of the osteoblast differentiation process (**B**). The bars indicate the percentage of positive cells in **A** (**C**) and in **B** (**D**). Data are expressed as mean ± SD obtained from 3 independent experiments.

The attachment and entry of HIV into susceptible cells depend on the presence of chemokine receptors CCR5 and CXCR4, along with CD4. In our study, we assessed the expression of these receptors on the surface of mesenchymal stem cells (MSCs) derived from the umbilical cord. Our findings revealed that 19.93 ± 2.23% of MSCs expressed CD4, 6.67 ± 0.39% expressed CXCR4, and 11.75 ± 0.78% expressed CCR5 (Figure 1A,C).

Subsequent experiments aimed to evaluate the potential modulation of HIV receptor expression during the differentiation of osteoblasts. The expression levels of CXCR4, CCR5, and CD4 were measured at 7 and 14 days post-differentiation because osteoblasts are exposed to the virus at these two time points. CXCR4 levels increased during the osteoblast differentiation process, reaching a peak at 14 days post-differentiation (38.5 ± 2.63%). The CCR5 expression level at 7 days post-differentiation was increased compared to MSCs (30.5 ± 2.26% vs. 16.10 ± 0.99%), but then (14 days) these levels significantly decreased, reaching values like those found in MSCs (16.1 ± 0.99%). In contrast, CD4 expression significantly decreased compared to undifferentiated cells (2.8 ± 0.28%) (Figure 1B,D). Together, these results indicate the HIV-receptor/coreceptors expression is modulated during the osteoblast differentiation process, thus influencing cellular susceptibility to virus attachment.

### 3.2. MSCs, but Not Osteoblasts, Are Abortively Infected by HIV

**Figure 2:**
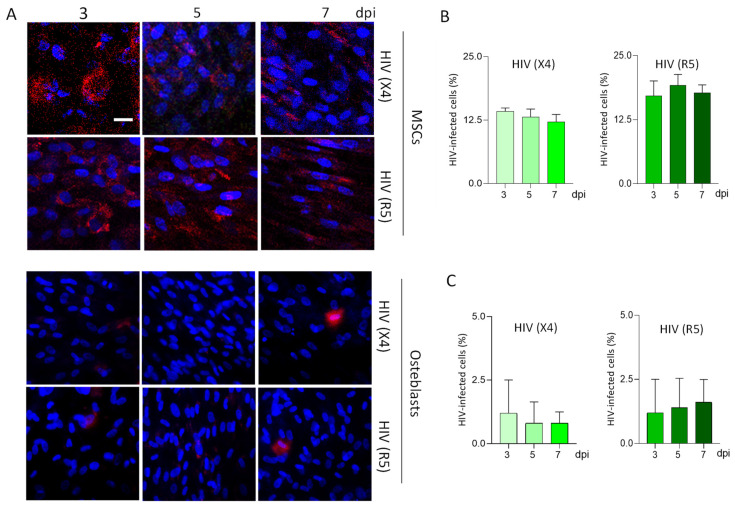
Exposure of MSCs and osteoblasts to X4- and R5-tropic HIV. MSCs and 14 days’ differentiated osteoblasts were infected with an inoculum of 1 pg of p24/cell with CXCR4-tropic HIV (HIV (X4)) and CCR5-tropic HIV (HIV (R5)). Representative microscopy images showing the kinetics of HIV replication at 3, 5, and 7 days post-infection (dpi) by the immunostaining of HIV-p24 capsid antigen (**A**). Bars express the percentage of HIV-infected MSCs (**B**) and osteoblasts (**C**). Ten microscopic fields per condition were quantified for each experiment. Scale bar: 50 µm. Data are expressed as mean ± SD obtained from 4 independent experiments.

Even showing dissimilar expression levels of CD4 and CXCR4/CCR5, MSCs and osteoblasts appear to be susceptible to HIV infection. However, their permissiveness was undefined. To ascertain, cells were exposed to R5-tropic HIV (AD8) or X4-tropic HIV (NL43) at an inoculum of 1 pg of p24 per cell for 24 h. Unbound viral particles were removed with several washes with culture medium. Viral infection efficiency in MSCs was assessed at 3, 5, and 7 days post-exposure using confocal microscopy and immunodetection, showing HIV capsid antigen expression and proviral DNA (via AluPCR). However, infection efficiency did not increase over time (Figure 2A,B), indicating that productive infection did not occur. In contrast, osteoblasts were susceptible but even less or not permissive to HIV infection (Figure 2A,C). Accordingly, only negligible capsid antigen expression was detected, and no proviral genome was found in osteoblasts. These results indicate that MSCs and osteoblasts do not produce HIV progeny. However, while MSCs can express HIV proteins and host the proviral genome, osteoblasts do not. This reflects that viral replication progresses more in MSCs than in osteoblasts. 

### 3.3. HIV Was Unable to Modulate Alkaline Phosphatase Activity in Osteoblasts 

The presence of diminished bone mass in patients who have not yet undergone treatment suggests that the virus independently impacts the equilibrium of bone health [5,6,32]. This allows us to hypothesize that HIV itself could affect osteoblast differentiation by infecting their MSC precursors. To test this hypothesis, MSCs were exposed to R5- and X4-tropic HIV in the presence of an osteoblast differentiation culture medium.

**Figure 3:**
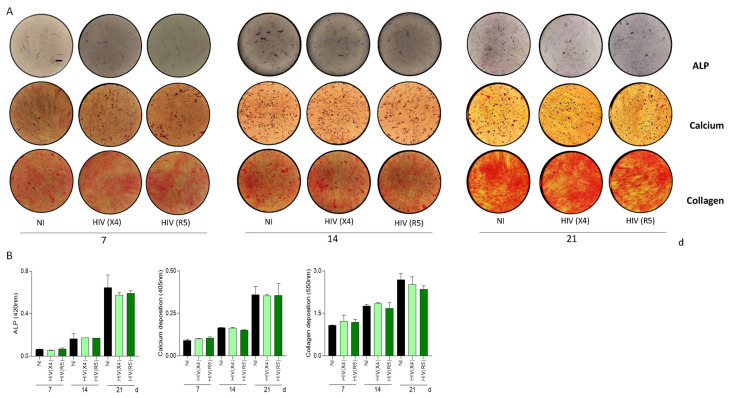
HIV was unable to modulate osteoblast differentiation. Effect of CXCR4-tropic HIV (HIV (X4)) and CCR5-tropic HIV (HIV (R5)) exposure on osteoblast differentiation. Representative microscopy images reveal alkaline phosphatase (ALP) activity by deposition of BCIP-NTB substrate, calcium deposition by alizarin red S staining, and collagen deposition by Sirius red staining at 7, 14, and 21 days post-differentiation (**A**). Spectrophotometric quantification of ALP activity, calcium, and collagen deposition (**B**). NI (noninfected). d (days post-differentiation). Ten microscopic fields per condition were quantified for each experiment. Scale bar: 200 µm. Data are expressed as mean ± SD obtained from 4 independent experiments.

Alkaline phosphatase (ALP) serves as both a crucial enzyme for mineralization and a marker for the osteoblast phenotype. Thus, ALP activity was measured at 7, 14, and 21 days during osteoblast differentiation. Our results indicated that R5- and X4-tropic HIV was unable to modulate ALP activity (Figure 3). These results indicate that HIV exposure was not able to modulate osteoblast differentiation and activity.

### 3.4. HIV Was Unable to Modulate Osteoblast Differentiation 

Osteoblasts are active cells with the responsibility of actively synthesizing bone. The predominant organic component of bone is composed of 90% type I collagen [33]. The correct deposition of the organic matrix is pivotal in enabling the proper placement of the mineral matrix and the formation of the architectural structure of bone [34]. This intricate process is characterized by the deposition and mineralization of the bone matrix, reinforcing the strength of the skeleton [35]. Then, experiments were carried out to investigate whether HIV infection could influence the deposition of both organic and mineral matrices. MSCs were subjected to infection with R5- and X4-tropic HIV in the presence of an osteoblast differentiation medium. The mineralization of osteoblasts was assessed at 7, 14, and 21 days post-differentiation by staining calcium-rich deposits with alizarin red S. Collagen deposition was examined through staining with Sirius red. Our results indicate that neither R5- nor X4-tropic HIV were able to modulate collagen and calcium deposition at 7, 14, and 21 days post-differentiation (Figure 3). Together, these results indicated that HIV exposure was unable to modulate matrix deposition by osteoblasts.

### 3.5. HIV Exposure Partially Alters RUNX2 Expression 

**Figure 4:**
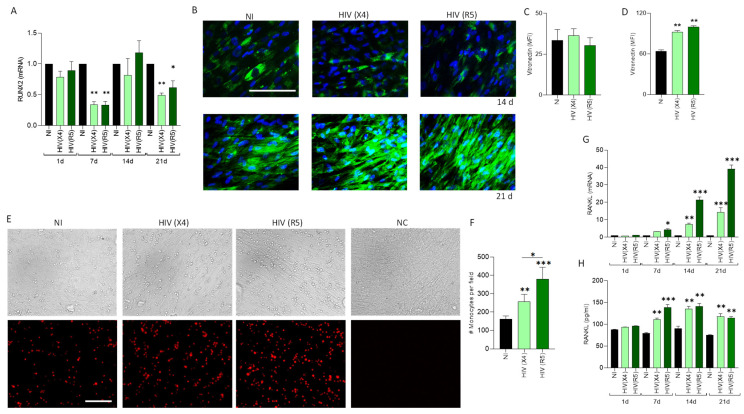
HIV modulates RUNX2, vitronectin, and RANKL expression during osteoblast differentiation. RUNX2 transcription was determined by RT-qPCR at day 1, 7, 14, and 21 post-differentiation (**A**). Vitronectin expression revealed by immunofluorescence with a specific antibody at 14 and 21 days post-differentiation (**B**). Quantification of median fluorescence intensity (MFI) using ImageJ from images in A at 14 (**C**) and 21 days post-differentiation (**D**). Adhesion of CellTrace^TM^ yellow-labeled U937 monocytes to differentiated osteoblasts previously infected or not with HIV-R5 or HIV-X4, uninfected osteoblast, and osteoblast without U937 (negative control, NC). The presence of adherent cells was determined by fluorescence microscopy (RED). DIC, differential interference contrast (**E**). Quantification of VPD-positive cells from images in **E** (**F**). *** *p* < 0.0001; ** *p* < 0.001; * *p* < 0.01 vs. non-infected (NI). RANKL transcription determined by RT-qPCR (**G**). RANKL expression in culture supernatants (**H**). NI (noninfected). d (days post-differentiation). Ten microscopic fields per condition were quantified for each experiment. Scale bar: 50 µm. Data are expressed as mean ± SD obtained from 3 independent experiments. * *p* < 0.01, ** *p* < 0.001, *** *p* < 0.0001 vs. NI.

Runt-related transcription factor 2 (RUNX2) is a transcription factor essential for initiating osteoblast differentiation and regulating the expression of bone formation markers [36,37]. Therefore, after exposing MSCs to HIV, the mRNA level of RUNX2 was measured during the differentiation process into osteoblasts at consecutive time points. Our results indicated that the RUNX2 gene mRNA level fluctuated throughout the differentiation process, with significant decreases observed at 7 and 21 days post-infection but recovery at 14 days post-infection (Figure 4A). These results indicate that HIV exposure transiently modifies RUNX2 transcription level in osteoblasts without visibly impairing mineral or organic matrix deposition.

### 3.6. HIV-Induced Vitronectin Expression from Osteoblasts 

Vitronectin is an abundant multifunctional glycoprotein found in the extracellular matrix of bone [38]. Osteoclasts are bone cells involved in bone resorption and express the vitronectin receptor αvβ3 integrin [39]. It is known that vitronectin induces osteoclasts to secrete tartrate-resistant acid phosphatase, which, in turn, promotes bone resorption [40]. This indicates that alterations in vitronectin expression could modify the balance of bone resorption. MSCs were differentiated into osteoblasts in the presence of X4- and R5-tropic HIV, and vitronectin expression was determined by fluorescence microscopy using immunodetection with a specific antibody at 14 and 21 days post-differentiation. Our results indicate that X4- and R5-tropic HIV induces a significant increase in vitronectin expression among osteoblasts (Figure 4B–D). These results indicate that HIV could alter bone metabolism by increasing vitronectin expression from osteoblasts that enhances osteoclasts’ resorption activity.

### 3.7. U937 Cells Adhere More to HIV-Infected Osteoblasts

Since vitronectin expression was upregulated in osteoblasts differentiated in the presence of HIV, we investigated their adherence to monocytes, as osteoclast precursor cells. To this end, osteoblasts were differentiated in the presence of X4- and R5-tropic HIV and then CellTrace^TM^ yellow uninfected U937 cells were added. Our results indicate that the percentage of adhered U937 cells, used as a model of monocytes, increased significantly when osteoblasts were differentiated in the presence of HIV (Figure 4E,F). Additionally, we observed that HIV-R5-tropic infection induces higher adhesion of monocytes compared to osteoblasts infected with X4-tropic HIV, despite no differences in the expression levels of vitronectin associated with viral tropism. 

### 3.8. HIV-Mediated RANKL Transcription among Osteoblasts 

The nuclear receptor activator ligand kappa B (RANKL) plays a crucial role in both the differentiation and activation of osteoclasts, thereby exerting a significant influence on bone remodeling [41]. Earlier studies have shown that this cytokine is upregulated in other instances of bone infections [42]. Thus, we investigated whether HIV (X4- and R5-tropic) infection of MSCs alters RANKL expression during osteoblast differentiation. Our results indicated that exposure to both X4- and R5-tropic HIV was able to upregulate the RANKL mRNA transcription level compared to noninfected cells at 7, 14, and 21 days post-differentiation but not at the early time point of 1 day (Figure 4G). Similar results were obtained when RANKL expression was evaluated in cell culture supernatants (Figure 4H). These results suggest that HIV-induced increases in RANKL expression among osteoblasts during their differentiation from MSC-infected precursors could enhance bone degradation by activating osteoclasts.

## 4. Discussion

PWH exhibit a higher prevalence of fractures compared to the general population. Consequently, the observation of low mineral density, often leading to osteopenia and osteoporosis, is common among them [1].

While osteoclasts play a crucial role in bone resorption, alterations in the differentiation of osteoblasts, cells responsible for the deposition of bone matrix, could also contribute to alterations in bone homeostasis [43]. 

MSCs are pluripotent cells capable of differentiating into various cell types, including osteoblasts. The susceptibility of MSCs to HIV infection was previously suggested based on mRNA transcription of HIV receptors and co-receptors [24,44,45,46,47,48]. However, documentation of cell surface receptor expression was lacking. Here, we show that MSCs express significant levels of CD4, CXCR4, and CCR5 on their surface. Conversely, during osteoblast differentiation, CD4 expression decreases to a minimum, while CXCR4 and CCR5 expression increase.

To elucidate MSCs viral permissiveness, in vitro studies have revealed that provirus is rarely found and productive infection was not documented [24,49]. Accordingly, in this manuscript, we demonstrated the susceptibility of MSCs to X4- and R5-tropic HIV infection but not their permissiveness to support productive HIV infection; however, proviral DNA and capsid-related antigens were detected, revealing a plausible abortive infection. In the case of osteoblasts, neither viral progeny nor proviral DNA was detected, in accordance with previous reports [19].

Aging and pathological conditions, including infections, can disrupt osteoblast differentiation and function [50]. This often accompanies concomitant bone resorption. Surprisingly, our results indicated that exposure of MSCs to both HIV tropisms did not alter mineral and organic matrix deposition during osteoblast differentiation. This finding contradicts previous studies suggesting that HIV inhibits osteoblast differentiation. However, those studies were conducted using human serum from individuals living with HIV with varying viral loads [46]; therefore, it is possible that the modulation involved not only the HIV load but also mediators present in the serum. Alternatively, some studies used HIV proteins to stimulate MSCs, an approach that may not necessarily mimic the effects of exposure to viral particles [17,44,51]. Meanwhile, the authors used a range of 0.1 to 1 ug of recombinant protein; this could simulate a massive infection similar to what is observed in untreated patients, as was previously reported [25,52]. 

During normal bone metabolism, osteoblasts and osteoclasts interact with each other, involving both their adherence and the production of vitronectin [38,39,53]. Here, we demonstrate that HIV infection of MSCs enhances both monocyte adherence and vitronectin production during osteoblastogenesis. Moreover, HIV-induced RANKL production but decreased RUNX-2 by osteoblasts creates a microenvironment that promotes increased bone resorptive activity by osteoclasts. Additionally, these monocytes could transdifferentiate into macrophages, which, in the context of HIV infection, may produce soluble mediators that modulate osteoblast differentiation and function [54].

Although HIV modulates RUNX expression, it does not lead to a modulation in the differentiation of MSCs into osteoblasts. We speculate that, since HIV does not modulate RUNX2 expression at 1 day post-differentiation, and despite a reduction at 7 days that is re-established by 14 days, RUNX2 expression is likely sufficient to commit osteoblasts at critical points. The reduction at 21 days post-differentiation seems less significant, as RUNX2 levels naturally decline during late osteoblast maturation [55,56,57]. Moreover, RUNX2 gene expression has been associated with age-related changes in bone mineral density [58]. This suggests that the transient decrease in RUNX2 observed in vitro could correlate with the early reduction in bone mineral density seen in PWH.

In this context, the increase in vitronectin and RANKL observed in response to HIV exposure appears to be controversial. Vitronectin, among other extracellular matrix proteins, interacts with membrane integrins to initiate intracellular signaling. This interaction triggers Runx2 phosphorylation and the expression of osteoblast-specific genes, suggesting a potential compensatory role for vitronectin [59]. On the other hand, RANKL expression can be influenced by Osterix (OSX). While RUNX2 regulates OSX, some studies suggest that OSX may also be regulated through alternate pathways during osteoblast formation, indicating that RANKL expression could be controlled by mechanisms beyond OSX [60,61,62]. Additionally, other compensatory mechanisms in response to reduced RUNX2 may be involved.

The differences in monocyte adhesion observed between osteoblast precursors infected with X4-tropic HIV and those infected with R5-tropic HIV could be attributed to the modulation of other adhesion molecules, among other factors [63]. Further studies are needed to elucidate these differences related to viral tropism.

In summary, the present study provides evidence that HIV modulates the differentiation process among abortively infected MSCs to osteoblast. Such a viral-mediated process impairs a critical step involved in bone homeostasis that may contribute to accelerating bone loss and dysregulated metabolism observed in PWH.

## Data Availability

The raw data supporting the conclusions of this article will be made available by the authors, without undue reservation.
